# The Effects of Retention Measures on Retirement Timing—Do Financial Crises Matter?

**DOI:** 10.3389/fsoc.2019.00068

**Published:** 2019-09-18

**Authors:** Tove Midtsundstad, Åsmund Hermansen

**Affiliations:** ^1^Fafo Institute for Labour and Social Research, Oslo, Norway; ^2^Department of Social Work, Child Welfare and Social Policy, Oslo Metropolitan University, Oslo, Norway

**Keywords:** companies' active aging policy, retention measures, effect studies, older workers, financial crises, fixed effects

## Abstract

The combined effect of rising life expectancy and declining fertility has made “aging” a dominant topic on the policy agenda across Europe. With the aim of retaining older workers and facilitating longer working lives, offering retention measures, such as the possibility of phased retirement, additional leave, and/or bonuses to older workers, has become a widespread strategy among Norwegian companies to combat voluntary early retirement. However, analyses do not find any overall effect of offering such retention measures, although some single measures like additional leave and bonuses seem to have reduced early retirement among older workers in Norway. The aim of this article is to examine whether the limited effect of companies' retention measures on early retirement have been impacted by the financial crisis of 2007/08. Our hypothesis is that the effect of companies' retention measures on early retirement will be less if companies were affected by the financial crisis of 2007/08. Although most companies affected by the crisis of 2007/08 still offers retention measures, the financial hardship following the crisis may force some to priorities to cut cost and reduce staff, which may in turn lead to earlier rather than delayed retirement among their older employees. In order to investigate whether the effects of retention measures on early retirement vary between individuals in companies affected by the financial crisis of 2007/08 or not, we use data from a survey carried out among a representative sample of Norwegian companies in 2010 combined with individual register data on all employees in these companies in the period 2000–2010. Using individual fixed-effects in combination with a linear probability model we did not find that the financial crisis of 2007/08 impact on retention measures overall effects on early retirement. However, working in a company affected by the financial crisis of 2007/08 seem to reduce bonuses and extra days offs' effect on early retirement among private sector employees; although the effects were not statistically significant. Hence it indicates, as expected, that the effect of retention measures on early retirement in the private sector are vulnerable to changes in companies' performance and the overall market situation.

## Introduction

The aim of the Norwegian employment policy is to promote high labor-force participation, low unemployment, and efficient labor-force utilization. In their efforts to increase employment among older workers, the Norwegian authorities have reformed the Norwegian pension system to increase the attractiveness of working after having reached the statutory retirement age. Moreover, through the initiation and signature of the agreement on a more inclusive working life (the IW Agreement), social partners have been assigned a more active role in the efforts to prevent early retirement and to increase the recruitment and retention of older workers. While the pension reform addresses the attractiveness of the pension system as a main cause for early retirement, and seeks to counteract early exit by strengthening the financial incentives targeting employees, the basic principle of the IW Agreement is that early retirement is an effect of workplace conditions and therefore needs to be counteracted by policies and initiatives targeting older workers in individual companies (Midtsundstad, 2015b).

Previous research on early retirement behavior has mainly been focusing on the impact of health and worker ability, working environment, and the design of the pension system on early retirement (the impact of the so-called push and pull factors). There has been much less focus on how factors related to companies' retention efforts impact on employment behaviors (Hasselhorn and Apt, 2015; Poulsen et al., 2017). Such measures or programs aimed at reducing early retirement and increasing employment of older workers can be categorized in different ways (Ilmarinen, 1999; Midtsundstad, 2005, 2011; Van Dalen et al., 2009). Midtsundstad (2011) distinguishes between strategies for prevention, retention, and integration. Retention efforts targets individuals who are already threatened with exclusion, or have access to an early retirement scheme, and have not the same scope and long-term impact as prevention programs. Instead, they will be for defined target groups. For example, initiatives to retain older workers in Norway before 2011 was often focused on employees around the age of 62 years who can retire on the contractual early retirement scheme (AFP scheme). With the aim of retaining older workers and facilitating longer working lives, offering retention measures, such as the possibility of phased retirement, additional leave and/or bonuses to older workers, became a widespread strategy among Norwegian companies to combat voluntary early retirement (Hermansen and Midtsundstad, 2015; Midtsundstad, 2015a,b). Analyses have, however, not find any overall effect of offering such retention measures (Midtsundstad et al., 2012a,b; Hermansen, 2015). Single measures, on the other hand, like additional leave, and bonuses seem to have reduced early retirement among older workers in Norway (Hermansen, 2014; Hermansen and Midtsundstad, 2018).

Generally, older workers have been among the least popular categories to recruit and retain in both periods of upturn and downturn (Ahmed et al., 2012; Baert et al., 2016; Carlsson and Eriksson, 2017), although employers have seemed more willing to retain than recruit older workers (Solem, 2015). Based on comparative surveys carried out among European employers in 2009, Conen et al. (2012) found that retention measures aimed at older workers was applied more in countries with low unemployment rates than in countries with higher unemployment rates. Furthermore, Conen et al. (2011) found in a study among Dutch employers that retention efforts increase when employers experience labor shortages. The same is found in Norwegian studies (Midtsundstad, 2005, 2011, 2015a). Hence, the labor market situation seems to affect employers demand for and willingness to retain older workers.

Van Dalen and Henkens (2013) have analyzed European employers' preferences and choices when threatened with prospect of mass lay-off of their employees as results of the Great Recession using survey data from six different countries (the Netherlands, Denmark, Germany, Sweden, Poland, and Italy). They found that employers in general prefer to tackle such threats by offering short-time work, and by early retirement packages to older workers, and buy-outs. However, the result was to some degree influenced by the strictness of the countries employment protection regulations. In countries where employers perceived the level of employment protection to be high the latter two strategies (early retirement and buy-outs) was their main preference. In addition, a general sense of generational fairness seems to influence employers' preferences. Those employers in favor of younger workers preferred early retirement and buy-outs when downsizing, followed by working time reduction. In other words, they preferred strategies that were mainly directed toward the older employees.

In line with this findings, Solem (2012), who analyzed the relationship between employers' attitudes to older workers and the financial crisis of 2008–2009 in Norway, based on data from CSP^1^'s Senior Policy Barometer from 2003 to 2009, found that the financial crises in 2008 and 2009 produced immediate reactions from managers. In particular, the protection of older workers during downsizing (i.e., the seniority principle) gained less support within the first half year of the crisis, especially in the private sector. However, there was a return to the former level of support within the next half year. The 2007/8 financial crisis did, however, affect Norway to a much lesser degree than other European countries. The most severe labor markets effects were observed in the oil and gas and off shore industry. However, it did only lead to a small increase in the unemployment rate among older workers, especially their long term unemployment (Midtsundstad, 2018).

Older workers themselves are also often inclined to “step down,” i.e., retire, in cases of redundancy, so that younger colleagues can keep hold of their current jobs (Midtsundstad and Bogen, 2011). A case study of eight different companies in four different industries in 2009–2010 (Midtsundstad and Bogen, 2011) found that this attitude is more prevalent among the lower-educated, such as industrial workers and care workers, than among highly-educated groups, such as engineers, and higher executive officers. They seem to feel that it is their moral obligation to retire, regardless of their own needs and wishes. This attitude may also affect older workers' willingness to make use of the companies' retention measures in order to continue working. The assumption that fewer jobs occupied by older workers will result in more jobs for younger workers is however unfounded (OECD, 2006). In a representative survey conducted in 2016 among Norwegian employees, 29% felt that it was the older workers who had to leave if the company has to reduce staff (Dalen, 2016a). In a parallel survey from 2016 among employers, 23% of managers shared this opinion (Dalen, 2016b). These views seem to be justified on the basis that older workers are, or soon will be, entitled to an old-age pension, or that it is easier for older workers to claim, and receive, a disability pension.

This empirical picture forms the backdrop to the research question addressed in this article. Our hypothesis is that the minor effects of companies' retention measures on early retirement may be affected by the financial crisis of 2007/08. It may be that the effect is lesser in companies affected by the crisis, because managers in such situations will have less financial room to offer special arrangements, and might then under-communicate the opportunity for such arrangements for older workers. In some cases, they will even have to reduce staff and offer their older employees early retirement and buy-outs, although the company still offer retention measures. Moreover, some of the older workers themselves will, we presume, under such circumstances may also be less willing to accept and make use of the companies special retaining arrangements and rather choose to retire; especially if the alternative is that younger workers have to leave the company.

We predict that as employers' demand for worker's changes, the demand for older workers also changes, and hence their efforts to retain older workers. At the same time, older workers' responsiveness to retention programs and measures also changes. Thus, the research question addressed in this article is*: to which degree are the effects of companies' retention measures on early retirement impacted by the financial crisis of 2007/08?*

## Data and Methodology

To investigate the impact of the financial crises, we use register data from the period 2000–2010 which comprises all 61-year-olds employed in one of the companies that participated in a 2010 survey. The company survey was conducted in the period August to September 2010 by Respons Analyse AS, a Norwegian research firm, on behalf of the Fafo Institute for Labor and Social Research. The company survey covers a representative sample of 800 Norwegian companies with 10 or more employees.

In Norway before 2011 the statutory retirement age was 67 years. Only employees in the public sector and about half of the employees in the private sector that worked in companies with a contractual early retirement pension (AFP scheme) had the opportunity to retire earlier, at age 62. In addition, some groups, mainly in the public sector, have a lower occupational retirement age (like nurses, assistant nurses, cleaners, bus drivers etc.) and can retire at age 62. Hence, to investigate the effects of companies' retention measures which are directed toward employees aged 62 or older we include only companies that offers an AFP scheme and employees aged 61 of older in the period 2000–2010. Of the 800 companies that participated in the 2010 survey, only 437 met these criteria−129 in the public sector and 308 in the private sector.

Selecting only companies with an AFP schemes means that the analyses only include companies and employees that are covered by collective agreements. As a result, the analysis will not be representative for older employees working in companies with low union density rates and coverage of collective agreements; in other words, most of the Norwegian service sector, like wholesale, retail, hotel and restaurant and the service industries.

The company survey provides a broad range of information on different company characteristics, such as sector, industry, managers' attitudes toward retention, whether the company was affected by the financial crises in 2007/8, whether they have signed the IW agreement, whether they had implemented an active-aging policy, whether they offered retention measures to retain older workers, what sort of measures they offered and what year the retention program was introduced, whether they find it hard or easy to offer different sorts of workplace and work-time adjustments etc. (see Midtsundstad and Bogen, 2011 for documentation of the survey). The company data are cross sectional, the data includes information on the year the retention measures were implemented, which enable us to identify older workers who were offered a retention measure when turning 62-year-old. We also know from the questionnaire that almost no companies changed their measures during the period analyzed in this article (Midtsundstad, 2014).

All information on the individual employees working in these companies was provided by Statistics Norway (SSB) and is drawn from administrative registers. We have information on gender, age, level of education, occupation, contractual working hours, income, debt, civic status, spouses' labor-market status, labor market status, withdrawal of pension benefits, etc.

The dependent variable is measured as withdrawal of a contractual pension in the next 2 years of employment, among workers still working at age 61 in the period 2000–2008. Thus, the dependent variable is measured in the period 2001–2010. Employees aged 61 and withdrawing early retirement benefits in the next 2 years of employment are given the value “1” upon withdrawal, whereas those who do not retire are given the value “0” on the dependent variable.

The retention measures are measured as dummy variables; those who are not offered any retention measure are given the value “0,” whereas those 61-year-olds who are offered a retention measure are given the value “1” when they turn 62. Thus, for those who are not offered any retention measures, the dummy variable is “0” at the ages of 61, 62, and 63, whereas for those who are offered a retention measure, the variable is “0” at age 61 and “1” at ages 62 and 63.

To investigate whether the retention measures offered have an effect on retirement behavior, we use the panel data method, individual fixed effects in combination with a linear probability model. The advantage of using individual fixed effects is that this model controls for all time-independent unobservable heterogeneity that could be correlated with the main independent variable and thereby produces an over- or underestimated coefficient. An individual fixed-effects model uses only variation within the same unit (individual) over time and therefore produces robust estimates on the effects of changes in different independent variables on different outcomes, controlling for all time-invariant explanatory variables and time-independent unobservable heterogeneity (Wooldridge, 2005, 2009; Angrist and Pischke, 2008). Estimating the effect of the retaining bonus using individual fixed effects and a linear probability model can be written as follows (Angrist and Pischke, 2008):

$$\begin{array}{l} y_{\text{it}}\;=\;\alpha_{i}\;+\;\lambda_{i}\;+\;\beta x_{it}\;+\;\beta 2TREAT_{it}\;+\;\beta 3AGE62_{i}\\ \;+\;\beta 4AGE63_{i}\;+\;\varepsilon_{it} \end{array}$$

where, *i* = 1… n, *t* = 1, …,*T*_*i*_.

In the analysis we investigate whether 61-year-olds working in companies offering a retaining measure at age 62 (β*2TREAT*_*it*_) have a lower probability of retiring early (y_it_) in the next 2 years of employment. β*2TREAT*_*it*_ equals “1” if the individual is offered a retaining measure at age 62, and is still “1” for these individuals at age 63. β*3AGE62*_*i*_ provides an estimate of the overall probability of retiring at age 62 for all included individuals and β*4AGE63*_*i*_ provides an estimate of the overall probability of retiring at age 63.

A company is identified as offering a retention program (intervention) if the HR manager, or executive director of the company in small companies, in August–September of 2010 reported to have such measures available to older workers with the purpose of encouraging them to continue working. Among the companies having introduced retention measures, only those with an entitlement age set at 62 years are included in the analysis.

To investigate whether the financial crises impact the effects the retention measures have on employees' retirement behavior, we conduct separate analyses for those working for companies where the HR manager/executive director reported in August-September 2010 that the company was highly or partly affected by the crises (value = “1”), and those employed by companies reporting to have been affected to a very small degree, or not at all, or that stated that they did not know whether they were affected or not (value = “0”).

As shown in Table 1 in the sample, 4,578, workers aged 61 over the period 2000–2010 works in a companies that were highly or partly affected by the crises in 2007/08, and 8,057 workers aged 61 over the period 2000–2010 works in companies that were affected to a very small degree, or not at all. When asked about the actual consequences for the company (Midtsundstad and Bogen, 2011), 30% of the managers reported that they had to cut company costs, 20% that they had to wait to recruit new employees, 13% that they had to reduce staff, either temporarily (11%) or permanently (11%). Only 3% reported that they had asked employees to draw an AFP pension early and only 2% stated that they had been forced to change or abolish some of the companies' retention measures.

**Table 1:** Descriptive statistics—control variables and key information for the group of 61-year-olds depending on the measure exposure (all sort of retention measures) and whether work in companies affected and not affected by the financial crises.

	**Affected by the financial crises**		**Not affected by the financial crises**	
	**Offered measures**	**Not offered measures**	**Offered measures**	**Not offered measures**
Women	61.4	53.1	61.1	66.7
Men	38.6	46.9	38.8	33.2
	100	100	100	100
Elementary school	24.0	30.6	15.5	16.4
High school	54.8	49.4	44.0	37.7
Undergraduate from university/college	14.6	15.6	28.3	35.8
Postgraduate from university/college	6.6	4.4	12.2	10.1
	100	100	100	100
Income percentile, mean	45.4	47.2	47.8	51.0
Spouse income percentile, mean	43.4	40.8	46.7	46.2
Household debts percentile, mean	40.3	37.6	41.0	42.2
Spouse retired on AFP retirement scheme	8.5	7.8	10.0	8.1
Spouse not retired on AFP	91.5	92.2	90.0	91.99
	100	100	100	100
Spouse retired on disability pension	16.6	19.1	16.0	14.0
Spouse not retired on disability pension	83.4	80.9	84.0	86.0
	100	100	100	100
Full-time 30 h or more	71.6	70.7	72.3	69.4
Part-time 20–29 h	12.7	13.3	12.4	14.5
Part-time <20 h	15.7	16.0	15.3	16.1
	100	100	100	100
Workers	57.1	57.7	49.0	38.0
Routine non-manual employee	18.3	20.3	17.7	41.7
Professional, administrator, or official	24.6	22.0	33.3	20.3
	100	100	100	100
Public administration	10.2	2.8	31.1	10.2
Teaching	8.85	13.6	22.1	44.9
Health and social services	15.8	21.2	29.3	27.7
Manufacturing	16.6	21.8	2.8	4.2
Construction	1.9	5.7	1.3	1.3
Hotels and restaurants	5.3	2.45	0.3	2.1
Wholesale and retail trade	7.9	13.3	2.5	3.2
Other industries	33.5	19.3	10.6	6.4
	100	100	100	100
*N*	2,124	2,454	2,213	5,844

In order to use a panel data method, some prerequisites have to be met. First, we have to be sure that the individuals studied are randomly distrusted between the intervention and the control group. In our case, it is up to each individual company to decide whether they want to offer retention measures or not. The group of Norwegian companies offering such programs/measures is therefore self-selected. However, in the analysis we use individual employee data, and the distribution of older workers in the intervention group and the control group can be assumed to be random, given that very few change jobs after the age of 60 in Norway (OECD, 2013). Moreover, those who do change jobs are often forced to do so, either because they have lost their current job, have reached the pension age limit, or because it is financially favorable to draw a pension and move to another employer, which many public employees do (Tofte et al., 2016). In other words, it is not very plausible that many older employees actively seek out, and move to, companies which offer special retention measures for older workers. Additionally, any time-invariant differences between workers being offered retention measures and those not receiving such offers, is controlled for when using individual fixed-effects methods. Furthermore, to control for any time-invariant differences, we have included time-variant controls in our analysis, guided by previous research (see below).

As individual fixed-effects control for all time-invariant explanatory variables and time-independent unobservable heterogeneity (Wooldridge, 2005, 2009; Angrist and Pischke, 2008) we only have to control for time variant risk factors. We therefore control for change in “income percentile” (measured as net income after tax divided into percentiles); “spouse income percentile” (measured as spouse income after tax divided into percentiles), “household debts percentile” (measured as household debts divided into percentiles). As earlier research shows that many couples “coordinate” their retirement (the “joint retirement hypothesis”) (Hank, 2004; Charles and DeCicca, 2007; Lancee and Radl, 2012), we also include the variables “spouse retired on the contractual pension” and “spouse retired on disability pension” in the analysis. Both “spouse retired on the contractual pension” and “spouse retired on disability pension” are measured as dummy variables given the value “0” if the spouse is not retired on the contractual pension or disability pension and “1” if the spouse retires on the contractual pension or disability pension.”

Table 1 presents the descriptive statistics for the group of 61-year-olds offered/not offered a retention bonus by companies affected/not affected by the financial crises in 2008.

As Table 1 shows, the sample comprises 4,578 individuals aged 61–62 who work/have worked for companies affected by the financial crises during 2008, and 8,057 individuals who work/have worked for companies not affected by the financial crises. The distribution of individual characteristics is fairly similar in the two groups when it comes to gender, income and debt, spouse's retirement, working hours and occupational group. The groups are dissimilar in regard to education level and industry; however, when using a fixed-effects model, these differences do not affect our results.

## Results

The results of the analyses are presented in Table 2.

**Table 2:** Individual probability of receiving an early retirement benefit among 62- and 63-year-olds, depending on the measure exposure (all sort of retention measures), before and after controlling for a range of individual time-variant variables.

	**All**		**Work for companies affected by the financial crises**		**Work for companies not affected by the financial crises**	
	**Model 1** **B** **(SE)**	**Model 2** **B** **(SE)**	**Model 1** **B** **(SE)**	**Model 2** **B** **(SE)**	**Model 1** **B** **(SE)**	**Model 2** **B** **(SE)**
Measure/Intervention	−0.000971 (0.0220)	−0.00089 (0.0210)	−0.0140 (0.0270)	−0.0107 (0.0256)	−0.0166 (0.0207)	−0.0137 (0.0198)
Age 61	0.141^***^ (0.0122)	0.137^***^ (0.0115)	0.187^***^ (0.0133)	0.181^***^ (0.0130)	0.123^***^ (0.0110)	0.121^***^ (0.0106)
Age 62	0.208^***^ (0.0161)	0.185^***^ (0.0146)	0.272^***^ (0.0164)	0.241^***^ (0.0158)	0.181^***^ (0.0143)	0.164^***^ (0.0133)
Income		−0.00467^***^ (0.000247)		−0.00484^***^ (0.000389)		−0.00447^***^ (0.000282)
Income spouse		−0.000770^***^ (0.000201)		−0.000521 (0.000356)		−0.000859^***^ (0.000245)
Household debt		−0.0000592 (0.000193)		−0.00017 (0.000259)		−0.000085 (0.000263)
Partner AFP		0.0615^***^ (0.0131)		0.1025^***^ (0.0285)		0.0421^***^ (0.0139)
Partner disability pension		0.0061 (0.0145)		−0.0077 (0.0229)		0.0127 (0.0188)
Observations	36,294	36,294	13,099	13,099	23,195	23,195
R-squared	0.161	0.194	0.205	0.237	0.137	0.140
Number of individuals	12,635	12,635	4,578	4,578	8,057	8,057

*For all, and for those working for companies affected and not affected by the financial crises. Both public and private sector*.

*** *p ≤ 0.01*.

According to our analyses, working for companies offering some sort of retention measures does not affect older workers' retirement behavior in itself. This corresponds to previous findings in earlier studies (Midtsundstad et al., 2012a,b). As our research question suggests, the lack of effect could be due to the financial crises, which might have impacted the companies' implementation and the managers' use of the retention measures available, as well as the older workers' take up of the measures offered.

However, we did not find any significant differences in the effects of the retention measures on retirement behavior either, when we compared those working for companies affected by the financial crises with those working for companies not affected by the financial crises.

One explanation might be that we did not run separate analyses for the different retention measures offered. Earlier studies have shown that those working in companies offering extra day offs and/or bonuses have an increased probability of postponing retirement (Hermansen, 2014; Hermansen and Midtsundstad, 2018), while reduced working hours has no effect (Hermansen, 2015). In light of this, we have analyzed the extent to which the effects of the different measures are dependent on whether or not the companies were affected by the financial crises. The results are presented in Table 3.

**Table 3:** Individual probability of receiving an early retirement benefit among 62- and 63-year-olds, depending on the measure exposure (extra days off and bonuses), before and after controlling for a range of individual time-variant variables.

	**Extra days off**				**Bonuses**			
	**Work in companies affected by the financial crises**		**Work in companies not affected by the financial crises**		**Work in companies affected by the financial crises**		**Work in companies not affected by the financial crises**	
	**Model 1** **B** **(SE)**	**Model 2** **B** **(SE)**	**Model 1** **B** **(SE)**	**Model 2** **B** **(SE)**	**Model 1** **B** **(SE)**	**Model 2** **B** **(SE)**	**Model 1** **B** **(SE)**	**Model 2** **B** **(SE)**
Measure/Intervention	−0.0454^***^ (0.0118)	−0.0406^***^ (0.0116)	−0.0372^***^ (0.0093)	−0.0318^***^ (0.0091)	−0.0032 (0.0143)	0.0001 (0.0122)	−0.0469^***^ (0.0140)	−0.0409^***^ (0.0122)
Age 61	0.2530^***^ (0.0071)	0.2464^***^ (0.0070)	0.2157^***^ (0.0049)	0.2098^***^ (0.0048)	0.2403^***^ (0.0065)	0.2342^***^ (0.0064)	0.2111^***^ (0.0046)	0.2060^***^ (0.0046)
Age 62	0.3730^***^ (0.0076)	0.3410^***^ (0.0077)	0.3281^***^ (0.0052)	0.3017^***^ (0.0052)	0.3570^***^ (0.0070)	0.3250^***^ (0.0070)	0.3230^***^ (0.0049)	0.2973^***^ (0.0049)
Income		−0.0051^***^ (0.0003)		−0.0057^***^ (0.0002)		−0.0052^***^ (0.0003)		−0.0057^***^ (0.0002)
Income spouse		−0.0006 (0.0004)		−0.0011^***^ (0.0003)		−0.0005 (0.0004)		−0.0011^***^ (0.0004)
Spouse AFP		0.1344^***^ (0.0224)		0.0857^***^ (0.0152)		0.1470^***^ (0.0214)		0.0905^***^ (0.0150)
Spouse disability pension		0.0004 (0.0249)		0.0290 (0.0187)		0.0022 (0.0242)		0.0339 (0.0189)
Observations	12,374	12,370	22,471	22,467	13,099	13,095	22,843	22,843
R-squared	0.277	0.304	0.245	0.277	0.274	0.302	0.244	0.276
Number of individuals	4,320	4,320	7,809	7,809	4,578	4,578	7,935	7,935

*For all, and for those working for companies affected and not affected by the financial crises. Both private and public sector*.

*** *p ≤ 0.01*.

The analysis in Table 3 shows that extra days off had an effect on the retirement behavior at age 62 and age 63 whether the employee works for a company affected by the financial crises or not. However, being affected by the financial crises or not does appear to have had an impact on the effects of bonuses on early retirement behavior. In companies not affected by the financial crisis of 2007/08 we find that being offered a bonus increases the probability of continuing working while there are no observable, statistically-significant effects on the retirement behavior of being offered a bonus among those working for companies that have been affected by the financial crises in 2008. However, there is an overlap between the confidence intervals in this two cases, so the differences in the effects are not statistically significant.

It is likely that the financial crises impact private sector companies more than public sector organizations. Hence, it might be that the impact of the financial crises on retention measures effect on retirement behavior is more visible among private sector employees than among public sector employees. We therefore perform a separate analysis for those employees working in private sector companies. The results are shown in Table 4.

**Table 4:** Individual probability of receiving an early retirement benefit among 62- and 63-year-olds, depending on the measure exposure (extra days off and bonuses), before and after controlling for a range of individual time-variant variables.

	**Extra days off**				**Bonuses**			
	**Work in companies affected by the financial crises**		**Work in companies not affected by the financial crises**		**Work in companies affected by the financial crises**		**Work in companies not affected by the financial crises**	
	**Model 1** **B** **(SE)**	**Model 2** **B** **(SE)**	**Model 1** **B** **(SE)**	**Model 2** **B** **(SE)**	**Model 1** **B** **(SE)**	**Model 2** **B** **(SE)**	**Model 1** **B** **(SE)**	**Model 2** **B** **(SE)**
Measure/Intervention	0.0195 (0.0174)	0.0138 (0.0171)	−0.0441^***^ (0.0169)	−0.0398^**^ (0.0167)	0.0533^**^ (0.0232)	0.0482^**^ (0.0228)	−0.1354^**^ (0.0538)	−0.1045^**^ (0.0530)
Age 61	0.271^***^ (0.0085)	0.2664^***^ (0.0084)	0.2380^***^ (0.0102)	0.2298^***^ (0.0084)	0.2587^***^ (0.0088)	0.2524^***^ (0.0086)	0.2238^***^ (.0098)	0.2163^***^ (0.0097)
Age 62	0.3997^***^ (0.0091)	0.3666^***^ (0.0093)	0.3657^***^ (0.0110)	0.3347^***^ (0.0112)	0.3861^***^ (0.0094)	0.3513^***^ (0.0097)	0.3544^***^ (0.0105)	0.3232^***^ (0.0108)
Income		−0.0046^***^ (0.0003)		−0.0049^***^ (0.0004)		−0.0046^***^ (0.0003)		−0.0051^***^ (0.0004)
Income spouse		−0.0000 (0.0006)		−0.0007 (0.0006)		0.0000 (0.0006)		−0.0007 (0.0007)
Spouse AFP		0.1384^***^ (0.0299)		0.0538 (0.0314)		0.1435^***^ (0.0152)		0.1449 (0.0152)
Spouse disability pension		−0.0056 (0.0309)		0.0588 (0.0358)		−0.0024 (0.0318)		0.0630 (0.0384)
Observations	8,439	8,437	5,914	5,916	7,621	7,621	5,376	5,376
R-squared	0.310	0.332	0.290	0.267	0.302	0.325	0.266	0.290
Number of individuals	2,953	2,953	2,074	2,074	2,976	2,976	2,097	2,097

*For all, and for those working for companies affected and not affected by the financial crises. Only private sector companies*.

** p ≤ 0.05,

*** *p ≤ 0.01*.

Although, we get almost the same results as when analyzing retirement behavior for both public and private sector employees together (Table 3), the separate analyses for public sector employees (Table 4) indicate that the financial crises have a stronger impact on the retention measures effect on the retirement behavior in the private than in the public sector. The results show that bonuses increase the probability of continuing working among employees in companies not affected by the crises, while it reduces the probability for continue working among those working in companies affected by the financial crises, and the difference is statistically significant. However, the results in Table 4 are uncertain due to the fact that there are very few private companies in the sample that both offers a retention measure and are affected by the financial crises.

Being offered extra days off also seem to increase the probability of continued working among employees in companies not affected by the financial crises, while it has no effect on the behavior among those working in companies affected by the financial crisis of 2007/08. However, these differences are not statistically significant.

## Discussion and Conclusion

To our knowledge, this is the first study investigating whether the effect of retaining measures on early retirement is affected by the financial crisis of 2007/08. According to our hypothesis, retention measures offered by companies affected by the financial crises might not reduce early retirement as measures offered by companies that were not affected by the crisis. This was tested by running separate analysis for individuals working in companies where managers reported that the company had been affected by the financial crisis of 2007/08 and for individuals working for companies where managers reported little or no effect of the financial crisis on the company's performance.

However, we did not find any significant differences in the overall effects of retention measures on early retirement behavior between the two groups. There was no effect of being offered a retention measure whether working in a company affected by the financial crisis or not. Neither did we find any difference in the effect on early retirement when analyzing the effects of the different single measures. The exception was for those working for companies offering a retention bonus: the effect of bonuses was strongest for those working in companies not effected by the financial crisis.

One explanation of the lack of observable impact of the financial crisis in general might be that older workers to a lesser degree than before are expected to leave companies that are experiencing financial hardship or have to reduce staff. If this is the case, the findings might indicate that there has been a change of attitudes both among managers and older workers themselves toward older workers. In other words, it might be that most Norwegian employers and employees do not hold the view any longer that it is the oldest employees that have to leave the company during times of financial hardship. This explanation is partly supported by Norwegian survey data from the period 2003–2016 showing that an increasing share of both managers and employees expect older workers to continue working to an older age than previously (Dalen, 2016a,b).

It is, however, likely that the financial crises impact private sector companies more than public sector organizations. When running separate analysis for public and private sector we also found that working in a company affected by the financial crisis of 2007/08 or not, seem to impact how bonuses and extra days off affect early retirement among private sector employees but not public sector employees. This indicate, as expected, that the effects of retention measures in the private sector are more vulnerable to changes in companies' performance and the overall market situation.

However, the differences in effects between private and public sector employees observed in this sample are not statistically significant. This may be due to the fact that there were very few private sector companies in the sample that both offered a retention measure and were affected by the financial crisis. In addition, the sample from private sector only covers companies with organized labor and collective agreements; hence the employees in the sample have a stronger dismissal protection than employees not covered.

Another possible explanation might be that the lack of, or limited effect of the retention measures offered is more a consequence of the nature of the types of measures offered than of unfavorable financial circumstances in some companies, which then obscures the measures' real effects on retirement behavior. It might be that most of the retention measures currently offered by Norwegian companies fail to meet the actual needs of older workers considering whether they should continue working or retire. This explanation has previously been discussed in other articles (Midtsundstad and Bogen, 2014; Hermansen, 2015; Midtsundstad, 2015b), where we, as an alternative to the emphasis on incentives, argue for the importance of a broader approach to active aging, in which the prevention of health problems and reduced work capacity is more emphasized. Instead of offering retaining bonuses or extra days off, which not affect the work situation or work ability of older workers, one might improve older workers' quality of work as a strategy for reversing the tendency toward early withdrawal from the labor market. Reducing health impairment, by improving working conditions and focusing on early prevention, may not only provide older workers with the opportunity to continue working, but also be an incentive to continue working by enhancing the desire to work longer and the belief in one's ability to do so (Hermansen and Midtsundstad, 2018).

## Data Availability Statement

The datasets generated for this study will not be made publicly available. The registry data used in combination with the survey data are on the loan from Statistics Norway and are not to be shared under Norwegian law.

## Author Contributions

TM developed the design of the study. ÅH prepared the data and completed the statistical analysis in collaboration with TM. TM and ÅH were involved in drafting the manuscript, interpretation of the results, and revising it critically for important intellectual content.

### Conflict of Interest

The authors declare that the research was conducted in the absence of any commercial or financial relationships that could be construed as a potential conflict of interest.

## Footnotes

^1^Center for Senior Policy, which is a Norwegian NGO promoting older workers' participation in the labor market.
